# Variation in smoking attributable all-cause mortality across municipalities in Belgium, 2018: application of a Bayesian approach for small area estimations

**DOI:** 10.1186/s12889-022-14067-y

**Published:** 2022-09-07

**Authors:** Polina Putrik, Martina Otavova, Christel Faes, Brecht Devleesschauwer

**Affiliations:** 1 Epidemiology, Maastricht University, Maastricht, the Netherlands; 2 Data Science Institute, I-BioStat, Hasselt University, Hasselt, Belgium; 3 Center for Demographic Research, UCLouvain, Louvain-la-Neuve, Belgium; 4 Department of Epidemiology and Public Health, Sciensano, Brussels, Belgium; 5 Department of Translational Physiology, Infectiology and Public Health, Ghent University, Merelbeke, Belgium

**Keywords:** Smoking prevalence, Smoking attributable mortality, Small area estimations, Bayesian hierarchical model

## Abstract

**Background:**

Smoking is one of the leading causes of preventable mortality and morbidity worldwide, with the European Region having the highest prevalence of tobacco smoking among adults compared to other WHO regions. The Belgian Health Interview Survey (BHIS) provides a reliable source of national and regional estimates of smoking prevalence; however, currently there are no estimates at a smaller geographical resolution such as the municipality scale in Belgium. This hinders the estimation of the spatial distribution of smoking attributable mortality at small geographical scale (i.e., number of deaths that can be attributed to tobacco). The objective of this study was to obtain estimates of smoking prevalence in each Belgian municipality using BHIS and calculate smoking attributable mortality at municipality level.

**Methods:**

Data of participants aged 15 + on smoking behavior, age, gender, educational level and municipality of residence were obtained from the BHIS 2018. A Bayesian hierarchical Besag-York-Mollie (BYM) model was used to model the logit transformation of the design-based Horvitz-Thompson direct prevalence estimates. Municipality-level variables obtained from Statbel, the Belgian statistical office, were used as auxiliary variables in the model. Model parameters were estimated using Integrated Nested Laplace Approximation (INLA). Deviance Information Criterion (DIC) and Conditional Predictive Ordinate (CPO) were computed to assess model fit.

Population attributable fractions (PAF) were computed using the estimated prevalence of smoking in each of the 589 Belgian municipalities and relative risks obtained from published meta-analyses. Smoking attributable mortality was calculated by multiplying PAF with age-gender standardized and stratified number of deaths in each municipality.

**Results:**

BHIS 2018 data included 7,829 respondents from 154 municipalities. Smoothed estimates for current smoking ranged between 11% [Credible Interval 3;23] and 27% [21;34] per municipality, and for former smoking between 4% [0;14] and 34% [21;47]. Estimates of smoking attributable mortality constituted between 10% [7;15] and 47% [34;59] of total number of deaths per municipality.

**Conclusions:**

Within-country variation in smoking and smoking attributable mortality was observed. Computed estimates should inform local public health prevention campaigns as well as contribute to explaining the regional differences in mortality.

**Supplementary Information:**

The online version contains supplementary material available at 10.1186/s12889-022-14067-y.

## Introduction

Smoking is one of the main causes of preventable mortality and morbidity worldwide. According to the World Health Organization (WHO), globally 12% of all deaths among adults aged 30 years and over were attributed to tobacco [1]. Tobacco use has a proven causal relationship with a number of chronic conditions, like cancer, cardiovascular disease, chronic respiratory disease and others [2]. In the COVID-19 pandemic, current smokers who contracted coronavirus were shown twice as likely to be hospitalized and tended to report more symptoms than non-smokers [3], although some controversy remains whether smokers are less likely to get infected with this virus [3].

Despite notable successful efforts by WHO and national governments worldwide to limit tobacco use [4], smoking prevalence in European countries remains a public health concern. Among the WHO regions, Europe has the highest prevalence of tobacco smoking among adults (28% in overall population, 38% in males and 19% in females) and one of the highest prevalence of tobacco use by adolescents [5]. It is thus not surprising that, compared to the rest of the world, the WHO European Region has one of the highest proportions of deaths attributable to tobacco use. WHO has estimated that tobacco use is currently responsible for 16% of all deaths in adults over 30 in European Region, with many of these deaths occurring prematurely [5].

Smoking prevalence in Belgium is slightly lower than the EU-15 average and was estimated at 15% in 2018. This proportion is higher in men (18%) than in women (12%). On the positive side, the prevalence of daily smoking has decreased by 40% between 1997 and 2018 [6]. Substantial regional variation in smoking rates was reported with higher prevalence in Wallonia (18%), followed by Brussels (16%) and Flanders (13%) and socio-economic disparities in smoking are persistent in Belgium similar to other countries [7–9]. Differences at lower geographical level (e.g. municipalities) may thus also exist while these have never been studied. These differences, if substantial, may be highly relevant for public health policies and prevention campaigns. Absence of municipality level statistics on smoking prevalence further hinders estimates of spatial distribution of smoking attributable mortality (i.e., the number of deaths that can be attributed to tobacco) in support of local health policy-making and understanding regional variations in mortality.

Since 1997, the Belgian Health Interview Surveys (BHIS) has been regularly collecting data on tobacco use in Belgium. The sampling design allows reliable estimates of many health indicators including smoking on national and subregional level, however, as only approximately one fourth of nearly 600 municipalities has been effectively sampled each survey round, direct estimates of smoking at municipality level are precluded. Recently, novel statistical methods have been proposed to address the growing demand for estimating small-area level indicators in situations when few or no data in some area precludes direct estimations [10]. In such cases, indirect estimates may be computed. Such estimates borrow strength from related areas through linking models based on auxiliary data such as recent census and administrative records [11]. Accounting for the complex survey design in computing these indirect estimates poses additional methodological challenges.

The aim of this study was to obtain estimates of smoking prevalence in each Belgian municipality using the BHIS 2018 data and to calculate smoking attributable mortality at municipality level using all-cause mortality statistics.

## Methods

### The Belgian Health Interview Survey (BHIS)

The BHIS is a state-funded cross-sectional population survey carried out every several years since 1997. In 2018, the sixth BHIS has been conducted. Stratified multistage clustered sampling is used to compose the sample: a limited number of municipalities are selected in which the survey is conducted. Within each municipality, a sample of households is drawn so that groups of 50 individuals are interviewed in total. Clustering also takes place at the household level since members of the same household are more alike than persons not belonging to the same household. To ensure that the final sample reflects the composition of the population, post stratification weights are calculated, considering population data from the National Register on age, gender, residence and household size.

### Study outcome and independent variables

For this study, data on smoking status (daily smoker, occasional smoker, former smoker and never smoked) and amount of cigarettes per day (categorized as “light smoking” in case of < 10 cigarettes per day, “moderate smoking” in case of 10 to 20 cigarettes per day, and “heavy smoking” for ≥ 20 cigarettes per day), age (in years), gender and educational level (no education/primary education, lower secondary education, higher secondary education, and highest education) of individuals aged 15 + were used. Statistics on age, gender and education level distribution for all Belgian municipalities were obtained from the 2011 census. The all-cause mortality data at municipality level in 2018 by age and gender were requested and obtained from Statbel.

### Statistical analyses

#### Direct and smoothed design based estimators for municipality level smoking prevalence

Our study interest is to estimate smoking prevalence per municipality using the BHIS data. A design-based direct and smoothed estimators are discussed in this section. This study deals with a binary outcome (smoking yes vs. no) denoted as an indicator variable ${\mathrm{y}}_{\mathrm{ik}}$ for the event of interest on the $\mathrm{k}$-th individual ($\mathrm{k}=1,\dots ,{\mathrm{N}}_{\mathrm{i}}$) in the $\mathrm{i}$-th municipality ($\mathrm{i}=1,\dots ,589$). The survey is conducted with sampling probabilities for person $\mathrm{k}$ from municipality $\mathrm{i}$ being ${\uppi }_{\mathrm{ik}}$. We denote by ${\mathrm{s}}_{\mathrm{i}}$ the set of individuals who are sampled from municipality$\mathrm{i}$. The design weights are an inverse of the sampling probabilities, i.e.${\mathrm{w}}_{\mathrm{ik}}= \frac{1}{{\uppi }_{\mathrm{ik}}}$. Horvitz-Thompson proposed the following formula for an estimator of prevalence ${\mathrm{P}}^{\mathrm{HT}}$[12]:

$$\mathrm P_{\mathrm i}^{\mathrm{HT}}={\sum\limits_{\mathrm{k{\in}s}_{\mathrm{i}}}\frac{{\mathrm y}_{\mathrm{ik}}}{{\mathrm\pi}_{\mathrm{ik}}}}$$ 

When the number of samples in each area is large and all areas are sampled, the design-based variance is usually small and Horvitz-Thompson estimates work well. However, in the BHIS, area sample sizes are small and samples are not available from each area, therefore smoothing over the areas may be considered. Bayesian hierarchical models offer a flexible approach to fitting such models and are used in this setting [12], in particular, a so-called Besag-York-Mollie (BYM) model that assigns a Conditional Autoregressive (CAR) distribution to the random effect to account for proximity of municipalities that share a common boundary [13–15]. A penalizing Complexity (PC) prior, using a scaled spatially structured component and an unstructured component [15] was used for the random effects. Compared to the default Gamma prior which is commonly used in BYM model, PC priors, as a weak information prior, have been suggested as useful, understandable and conservative [15]. A sensitivity analysis with alternative parameters of the PC prior assigning more prior mass on smaller variance of the random effects were conducted to assess the robustness of results to the choice of the prior. Further details can be found in Supplementary file 1 (Technical details on design-based estimators).

To take into account the survey design, the logit transformation of the design-based direct estimates of the prevalence was modelled as $\widehat{\mathrm{p}}$_i_ ~ N(${\uptheta }_{\mathrm{i}}$_,_$\widehat{{\mathrm{V}}_{\mathrm{i}}}$), where ${\uptheta }_{\mathrm{i}}$ is as specified above and $\widehat{{\mathrm{V}}_{\mathrm{i}}}$ is the asymptotic variance on the logit scale. A series of models including combinations of area-level covariates (age, gender and education distribution of the population at municipality level) were fitted. We estimated prevalence for two indicators, namely, current smoking vs non-current smoking (former or never), and ever smoking (current and former) vs never smoking. Prevalence and 95% credibility interval of former smokers were obtained by bootstrapping as difference between the prevalence of current and former smokers and prevalence of former smokers. Similarly, estimates of heavy, moderate, light, former and never smoking were obtained. Differences computed during bootstrapping were bounded to be positive (i.e., negative differences were set to zero and respective prevalence estimates were rescaled so that sum of categories remained equal to 100%). For the purpose of these analyses, individuals with missing data (19%, see Supplementary file 1 (Table S1) for details) on smoking status were excluded given the methods have not yet been developed to be applied to multiple imputed data.

BYM belongs to a family of Bayesian hierarchical models. Bayesian inference is useful to model the spatial and spatio-temporal data as it allows for complete flexibility in how estimates borrow strength across space and time and allow to account for similarities based on sharing borders or on the distance [13, 16]. Integrated Nested Laplace Approximation (INLA) was used to obtain estimates of model parameters. INLA has been shown as a more efficient and less time-consuming alternative to Markov chain Monte Carlo (MCMC) methods.

Fitted models were assessed and compared using the deviance information criterion (DIC) and the conditional predictive ordinates (CPO). The DIC is a criterion based on the trade-off between the fit of the data to the model and the corresponding complexity of the model. The CPO is the probability density of an observed response based on the model fit to the rest of the data. Large values indicate a better fit of the model to the data, while small values of the CPO represent unexpected response values. CPOs were plotted and inspected visually.

### Smoking attributable all-cause mortality

#### Relative risks for all-cause mortality from smoking

Ovid Medline was searched (02/02/2021) for published systematic reviews of original studies of relative risk (RR) estimates for all-cause mortality related to smoking using filters for publication type (“systematic review”) and a combination of search terms “smoking” and “all-cause mortality”. The following information was extracted from eligible studies: author and date of publications, region/country of included studies, age and gender characteristics of study population, and RR risks for all relevant exposures.

#### Population attributable fraction and smoking attributable mortality

The population attributable fraction (PAF) is the proportion of cases of an outcome of interest that can be attributed to a given risk factor among the entire population [17]. When several categories of exposure to the risk factor exist, the formula takes the form:

$${\mathrm{PAF}}_{\mathrm i}=\frac{\sum_{\mathrm j=1}^{\mathrm n}{\mathrm P}_{\mathrm i}\left(\mathrm{RR}-1\right)}{\sum_{\mathrm j=1}^{\mathrm n}{\mathrm P}_{\mathrm i}\mathrm{RR}},$$ 

where $\mathrm{i}=1,\dots ,\mathrm{k}$ is the number of municipalities, and $\mathrm{j}=\mathrm{1,2},\dots ,\mathrm{n}$ is the number of exposure categories.

The PAF is usually expressed as the percentage of disease cases/deaths attributable to exposure. Smoking attributable mortality (SAM) can be obtained as.

$${\mathrm{SAM}}_{\mathrm{i}}$$ = $${\mathrm{D}}_{\mathrm{i}}$$ * $${\mathrm{PAF}}_{\mathrm{i}}$$,

where ${\mathrm{D}}_{\mathrm{i}}$ is the number of observed deaths in municipality $\mathrm{i}$.

Smoking attributable mortality was calculated using two scenarios. In a first scenario, we accounted for the differences in population demographic structure between municipalities by performing calculations using the number of deaths and smoking prevalence standardized to the age and gender distribution of total Belgian population. In a second scenario, given that prevalence of smoking behavior varies between age and gender groups, the calculations were performed in six strata by age and gender (age 15–39, 40–64, 65 + , males and females) and subsequently summed up to a total number of deaths per municipality that can be attributed to smoking.

In each scenario, mean estimates and 95% credibility intervals estimates were obtained by performing 1000 computations based on 1000 samples from the posterior distribution of smoking prevalence and 1000 samples from gamma distribution assumed for the relative risks. Number of computations was determined empirically, i.e. increasing number of computations from 500 to 1000 resulted in minor changes in computed estimates therefore 1000 computations were considered sufficient. Gamma distribution was chosen given its properties are compatible with the expected shape of the underlying distribution of RR: it is continuous, non-negative and puts relatively less weight on the tails of the distribution compared to e.g. log-normal or uniform distribution.

All analyses were conducted in R (R version 4.0.4, R studio version 1.4.1103). Smoothed estimates were obtained with SUMMER package (version 1.1.0) [18, 19].

## Results

### Descriptive statistics

**Fig. 1:**
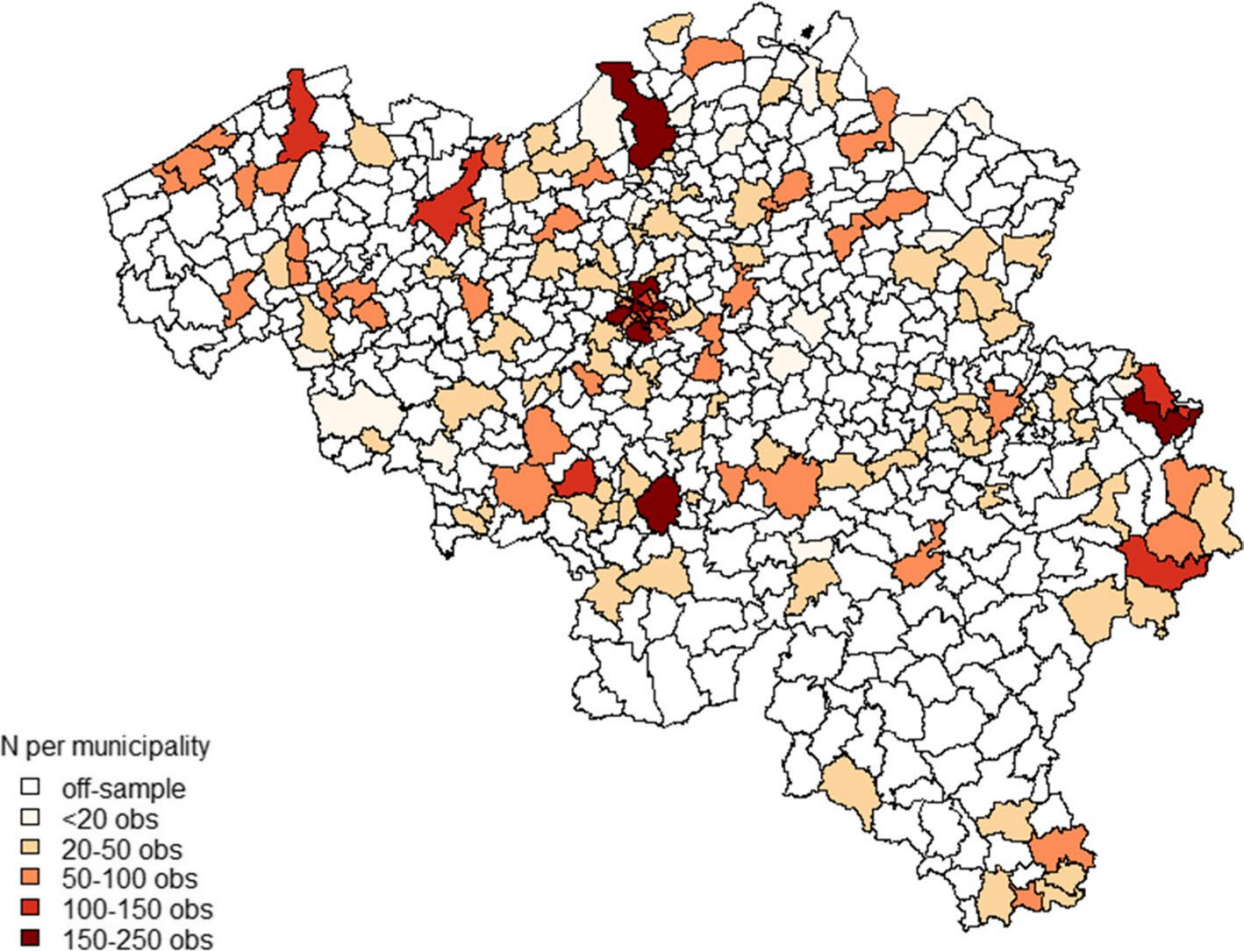
Belgian Health interview survey: in- and off-sample municipalities

The BHIS 2018 data included 9,753 respondents from 154 municipalities with 7,829 included in the analyses. The number of observations per municipality varied from 2 to 249 (Fig. 1). The mean age of participants (accounting for survey weights) was 48 years and 48% were males. Half of the respondents (50%) had high education and one third (32%) had secondary school diploma. Fifteen percent were daily smokers, 4% smoked occasionally, 23% reported to have quit smoking and 58% have never smoked. Heavy smokers constituted 2% of the total population, with another 10% being moderate smokers and 7% light smokers (Table 1). Proportion of missing data in smoking status was higher among lower educated individuals, with no differences by age or gender (23% vs 16%, see Supplementary file 1 (Table S1) for details).

**Table 1:** Survey variables –weighted estimates, based on 7,829 respondents

Variable	Weighted estimates
Age, mean (SD) years	48.86 (0.34)
Age groups, N (%)[95%CI]	
15–39 y.o	2 581 983 (34%) [2 428 208; 2 735 758]
40–64 y.o	3 285 758 (44%) [3 136 226; 3 435 289]
65 + y.o	1 677 012 (22%) [1 575 160; 1 778 864]
Gender, males N (%)[95%CI]	3 655 177 (48%) [3 522 770; 3 787 584]
Education, N (%)[95%CI]	
No education or low school diploma	389 829 (5%) [336 169; 443 489]
Low education	892 111 (12%) [802 728;;981 494]
Secondary education	2 423 262 (32%) [2 265 392; 2 581 132]
High education	3 747 714 (50%) [3 556 339; 3 939 090]
Smoking status, N (%)[95%CI]	
Daily smoker	1 161 611 (15%) [1 068 527; 1 254 695]
Occasional smoker	301 872 (4%) [255 408; 348 336]
Former smoker	1 739 813 (23%) [1 635 811; 1 843 814]
Never smoked	4 341 457 (58%) [4 169 599; 4 513 315]
Smoking status with by level of exposure, N (%)[95%CI]	
Light smoker (< 10 cig/day)	533 995 (7%) [473 811; 594 179]
Moderate smoker (10 to 20 cig/day)	775 460 (10%) [697 827; 853 093]
Heavy smoker (≥ 20 cig/day)	154 028 (2%) [121 308; 186 748]
Former smoker	1 739 813 (23%) [1 635 811; 1 843 814]
Never smoked	4 341 457 (58%) [4 169 599; 4 513 315]

### Design-based and smoothed municipality level prevalence of smoking

**Fig. 2:**
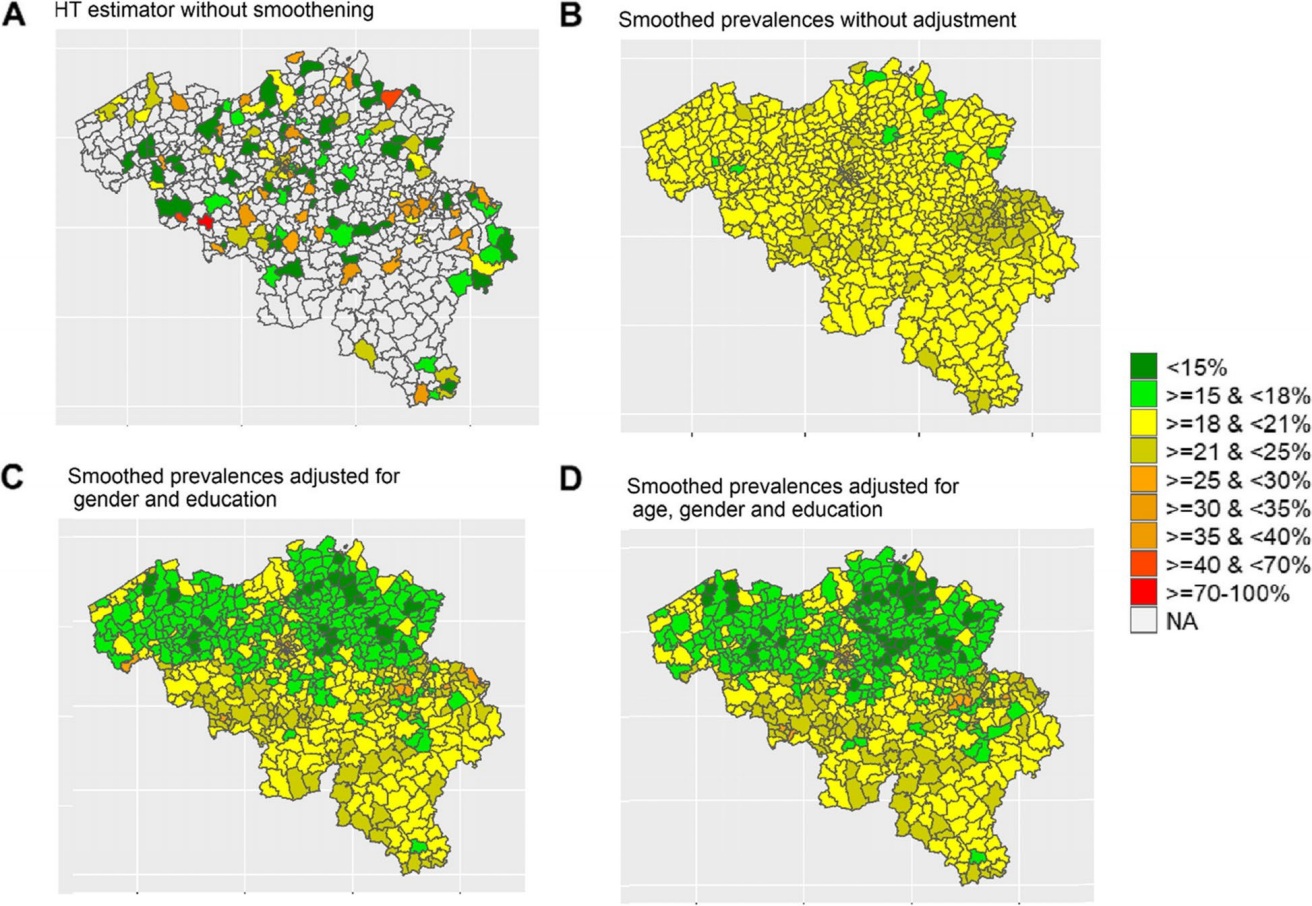
Prevalence of smoking behavior (current smoker) per municipality. **A** Horvitz–Thompson estimator design-based estimates, **B** Smoothed estimates without adjustment for covariates, **C** Smoothed estimates adjusted for education and gender at municipality level, **D** Smoothed estimates adjusted for education, gender and age at municipality level

The design-based Horvitz-Thomson estimator of smoking prevalence for in-sample municipalities varied between 0 and 100% (Fig. 2 A). This large variation can be attributed in particular to small sample sizes in many municipalities. Smoothed prevalence obtained from the hierarchical model without adjustment for area-level demographic and socio-economic covariates revealed less variability in smoking prevalence between municipalities (Fig. 2 B). The DIC of this model (254.08) however revealed worse fit compared to models that accounted for socio-demographics (DIC 248.23 for a model with gender and education). Adjusting for municipality level covariates (education, gender and age) further calibrated the estimates of smoking prevalence (Fig. 2 C & D). Smoothed estimates for current smoking ranged between 11–27% per municipality, and for former smoking between 4 and 34% (Table 2). Sensitivity analyses with alternative parameters for the PC prior showed that the model was robust to the choice of priors (Supplementary file 1 (Fig. S1).

**Table 2:** Smoothed prevalence of current (heavy, moderate and light smokers), former smokers and persons who never smoked with 95% credible interval from model adjusted for municipality level age, gender and education

**Municipality**^a^	**Current smokers**				**Former smokers**	**Never smoked**
	Heavy	Moderate	Light	Total current		
Antwerpen	0.023 [0.012;0.036]	0.087 [0.057;0.119]	0.092 [0.042;0.143]	0.202 [0.161;0.245]	0.225 [0.150;0.300]	0.573 [0.510;0.635]
Meise^b^	0.036 [0.016;0.063]	0.054 [0.014;0.095]	0.063 [0.002;0.127]	0.153 [1.103;0.209]	0.214 [0.071;0.366]	0.633 [0.485;0.763]
Gent	0.024 [0.010;0.043]	0.055 [0.024;0.086]	0.095 [0.045;0.148]	0.173 [0.131;0.220]	0.238 [0.148;0.329]	0.588 [0.508;0.666]
Liège	0.028 [0.014;0.045]	0.088 [0.049;0.132]	0.100 [0.030;0.172]	0.216 [0.159;0.279]	0.231 [0.113;0.349]	0.553 [0.449;0.651]
Genk	0.057 [0.014;0.122]	0.074 [0.000;0.145]	0.051 [0.000;0.137]	0.182 [0.119;0.254]	0.232 [0.092;0.375]	0.586 [0.457;0.705]
Hasselt	0.031 [0.016;0.050]	0.049 [0.019;0.081]	0.064 [0.014;0.116]	0.144 [0.102;0.191]	0.176 [0.066;0.292]	0.681 [0.573;0.782]
Lanaken^b^	0.056 [0.017;0.113]	0.078 [0.002;0.144]	0.045 [0.000;0.129]	0.178 [0.115;0.251]	0.247 [0.081;0.417]	0.575 [0.416;0.724]
Rendeux^b^	0.035 [0.012;0.070]	0.110 [0.050;0.171]	0.072 [0.000;0.166]	0.217 [0.147;0.296]	0.240 [0.072;0.409]	0.542 [0.387;0.691]
Herbeumont^b^	0.043 [0.021;0.071]	0.116 [0.065;0.169]	0.057 [0.000;0.138]	0.217 [0.156;0.284]	0.256 [0.096;0.418]	0.527 [0.374;0.673]

^a^ Estimates for all 589 municipalities are provided in Supplementary Table S2

^b^ off-sample municipalities

Comparing design-based estimators with smoothed prevalence estimates (for the 154 in-sample municipalities) revealed a great level of smoothing, in particular for municipalities with few observations (Supplementary file 1 Fig. S2). CPO diagnostics of fully adjusted model revealed few municipalities with very low values (Supplementary file 1 Fig. S3). Data check revealed that these were the municipalities with very high (e.g. 46% in municipality of Mechelen) or very low (e.g. 7% in municipality of Maasmechelen) design-based smoking prevalence which may be attributed to random variability due to small number of observations. These estimates were namely smoothed to 19% and 18% for Mechelen and Maasmechelen, respectively.

### Smoking attributable all-cause mortality

**Fig. 3:**
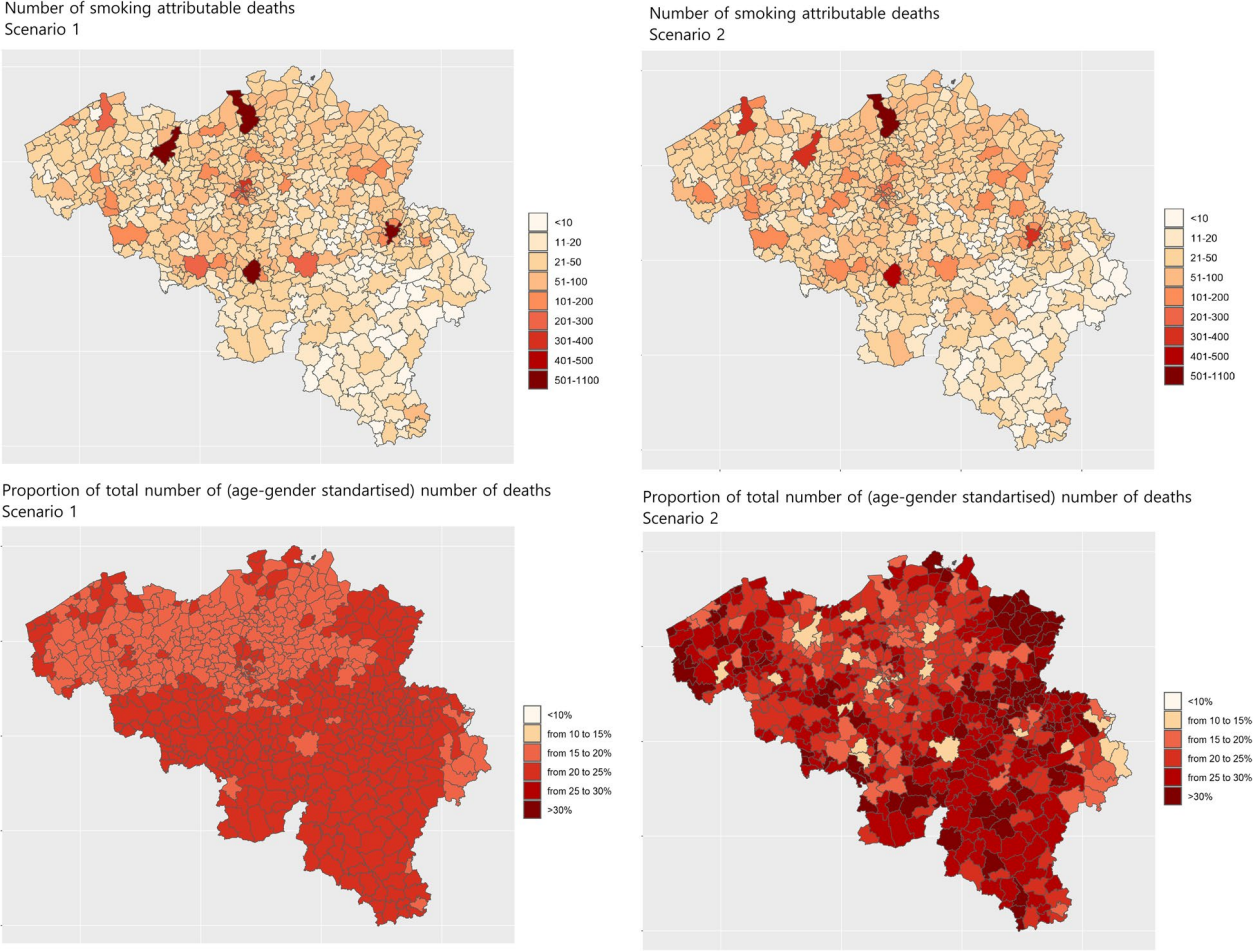
Smoking attributable mortality (as absolute number of deaths and proportion of total deaths) according to the two scenarios

The literature search yielded 74 papers, of which four meta-analyses provided pooled estimates of relative risk for all-cause mortality from smoking (Table 3). Smoking attributable mortality calculated according to the two outlined scenarios is presented in Table 4 and Fig. 3. Different assumptions made across the scenarios resulted in differences in number of deaths attributable to smoking indicating the uncertainty inherent to such estimates. In terms of fraction of total number of deaths, smoking was responsible for between 10 and 47% of total mortality in municipalities.

**Table 3:** Relative risks for all-cause mortality from smoking

Study	Region/ country	Gender	Age	Exposure	RR (95% CI)	Years of follow-up	Adjustment for confounders
Jacobs 1999 [20]	Europe, US, Japan	M M	40–59 40–59	Light (< 10 cig/day) vs never > = 10 cig per day vs never	1.30 (1.20 – 1.40) 1.80 (1.70 – 1.90)	25-years	Baseline country of residence, age, body-mass index, serum cholesterol, SBP and clinical CVD
Gellert, 2012 [21]	US, China, Aus, Japan	M/F M/F	60 + 60 +	Current smoker vs never Former smoker vs never	1.83 (1.65 – 2.03) 1.34 (1.28 – 1.40)	3 – 50 years	Subgroup analyses by age, and region of study conduction
Carter 2015 [22]	US	M F	55 + 55 +	Current smoker vs never Current smoker vs never	2.80 (2.80 – 2.90) 2.80 (2.70 – 2.90)	11 years	Age, race, educational level, daily alcohol consumption, and cohort
Shavelle 2008 [23]	US, Asia, Europe	M/F	Adults (mean age in included studies 30–65 +)	Light (< 10–21 cig/day)^a^ vs never Medium (10–25 cig/day)^a^ vs never Heavy (20 + /25 + cig/day)^a^ vs never Former vs never	1.47 (1.37 – 1.80) 2.02 (1.84 – 2.36) 2.38 (2.17 – 2.84) 1.31 (1.07 – 1.39)	NR	NR

^a^ Used definition by authors, which was not consistent thus categories overlap. Review authors conducted sensitivity analyses and concluded that results were robust to category definitions; *M* Male, *F* Female, *SBP* Systolic blood pressure, *CVD* Cardiovascular disease, *NR* Not reported

**Table 4:** Smoking attributable all-cause mortality per municipality, in absolute number of deaths

**Municipality**^a^	**Number of smoking attributable deaths in 2018** **Estimated number [best–worst case scenario], % of total deaths**^c^	
	Scenario 1	Scenario 2
Antwerpen	1067 [819; 1344] (18%)	779 [472; 1190] (16%)
Meise^b^	38 [25; 48] (20%)	45 [13; 106] (25%)
Gent	535 [390; 671] (19%)	354 [175; 656] (15%)
Liège	535 [406; 654] (21%)	377 [188; 673] (17%)
Genk	166 [115; 213] (22%)	116 [48; 232] (17%)
Hasselt	141 [99; 185] (19%)	131 [58; 260] (17%)
Lanaken^b^	53 [38; 65] (22%)	83 [23; 142] (34%)
Rendeux^b^	5 [4; 6] (22%)	6 [2; 11] (30%)
Herbeumont^b^	2 [2; 3] (21%)	2 [1; 4] (24%)

Number of deaths is rounded up

^a^ Estimates for all 589 municipalities are provided in the Supplementary Table S3

^b^ off-sample municipalities;

^c ^age-standardized number of deaths in scenario 1 and crude number of deaths in scenario 2

## Discussion

The objective of this study was to apply the Bayesian hierarchical model to obtain indirect small area estimations of smoking prevalence in Belgium and compute municipality-level smoking attributable mortality. The final estimates of current/former smoking prevalence ranged between 11 [Credible Interval 3;23] and 27% [21;34] between the municipalities. Across Belgian municipalities, smoking was assessed to be the cause of 10 [7;15] to 47% [34;59] of all deaths. This demonstrates that smoking poses a serious public health burden and, given that smoking-related deaths are avoidable, it also presents a prevention opportunity to improve quality and duration of life of many people.

To our knowledge, this is the first study to compute municipality level estimates for smoking and smoking attributable mortality in Belgium. Information about spatial distribution of these health indicators should support local policy-making as well as contribute to explaining the regional differences in mortality. The methodology used in the paper can be broadly applicable in the context of national health surveys in other countries.

While a number of small-area estimation methods have been proposed in the literature, it was out of scope of this work to provide a comprehensive comparative analysis of these methods and we rather focused on practical application of the method proposed by Mercer et al. [12]. This approach had the advantage in a sense that it is suitable for complex survey design with large amounts of off-sample areas. A crucial question is the validity of the estimates in the off-sample areas as well as in areas with very few respondents.

With respect to off-sample areas and in an absence of the true estimates in each municipality it is hard to make strong claims about the validity of the produced estimates. Existing methodological literature including simulation studies suggests this method yields valid estimations [12], however, it is reasonable to expect for its accuracy to decrease with increasing number of off-sample areas. In the current setting, data were available from 154 of 589 municipalities, which implied a lot of smoothing and extrapolation. To our knowledge, no simulation studies are available to assess the performance of this method in context where almost three quarters of the areas were off-sample (Watjou et al. have omitted up to 56% of the areas in their simulation study [10]). To improve the accuracy of estimations, auxiliary data from three municipality-level socio-economic covariates (age, gender and education) were used. These factors have been shown to be associated with smoking in the BHIS data but also from previous research [24], and thus, accounting for distribution of municipality population across age, gender and educational groups was expected to further fine-tune the estimates. In fact, only minor improvements in the fitted models were observed according to DIC which may be explained by ecological fallacy – area-level covariates may fail to pick up the existing associations with the outcome on individual level. Incorporating individual level covariates in the model would likely improve the predictions; however, more methodological research is needed to accommodate challenges related to complex survey data (e.g. how to incorporate the individual weights when part of areas is off-sample) and missing data (e.g. missing outcome or covariate data in survey data and in off-sample areas) in this context. In current analyses, provided estimates of smoking prevalence are likely conservative given lower educated respondents (how are known to be more frequently smokers) were more likely to leave the question on smoking blank. For missing survey data multiple imputation (MI) could provide a remedy under assumption of ‘missing at random’ (MAR) [25]. Smoothed estimates method should be further developed to accommodate the theoretical and practical implementation of MI in this context. Last but not least, given the increasing policy relevance of small area estimates, future rounds of the BHIS survey may reconsider the tradeoff between increasing the number of in-sample areas, which improve possibilities of small area analyses, and the cost implications of such design changes.

With respect to areas with very few respondents, model diagnostics revealed substantial smoothing towards the mean prevalence. Where direct smoking prevalence estimates (based on 2 to 249 respondents) ranged between – likely unplausible – 0 and 100%, the smoothed indirect estimates had a range of 11 to 27%. This is subject to large uncertainty if the sample size is very small. On the other hand, omitting important covariates can result in over smoothing, so obtained estimates should be interpreted with caution and validated in future studies.

Another important methodological step in this work was calculating the population attributable fractions that are subsequently used to obtain smoking attributable mortality. These widely used in public health measures are subject to strong limitations. First, these calculations rely on assessment of relative risk of smoking for all-cause mortality from existing studies across the world or indirect methods such as Peto-Lopez method that bases relative risks on the excess rates of lung cancer mortality [26]. For this study, we have used the estimates from four meta-analyses of existing studies [20–23] as meta-analyses provide more robust and stable estimates of relative risks compared to single studies. However, the obtained estimates did not cover in sufficient detail all age and gender groups of general adult population. Analyses under the two scenarios (standardizing the population for age and gender vs summing up estimates from six strata's by age and gender) resulted in a wide range of estimates with wide confidence intervals reflecting substantial uncertainty (Table 4). Second, while relative risks were obtained after adjusting for several important confounders, the calculations of population attributable fraction may still fail to account for a number of possible exposures such as, for example, air quality or work-related exposure. Third, important considerations were made in respect of standardization of number of deaths per municipality to age and gender structure of general Belgium population. On the one hand, estimates of relative risks may have already captured the age and gender distribution in the study cohort (which may or may not be comparable to Belgian population), on the other hand, obtaining PAF estimates on standardized or stratified number of death per age and gender group was supposed to explicitly eliminate differences in number of deaths between relatively ‘green’ (with large proportion of young population) and ‘grey’ (with large proportion of older population) municipalities. To further improve the accuracy of PAF calculations, data on age and gender specific relative risks of all-cause mortality from smoking in Belgian population are needed. Last but not least, the estimates of smoking prevalence and death statistics from 2018 were used, while there is a known large lag between the onset of smoking, length of exposure to smoking and death. Given that smoking rates have been declining substantially over the last 20 years [6], provided estimates of smoking attributable mortality are likely to be conservative as deaths occurring in 2018 are related to past smoking.

In conclusion, substantial within-country variation in smoking and subsequently smoking attributable mortality was observed. This study provided an illustration of the methodological approach to obtain small area estimates from national health survey that can be used for a broad range of health indicators.

## Supplementary Information

**Additional file 1: **Technical details ondesign-based estimators.**Table S1.** Proportion of missing data on smoking by education, age and gender. **Figure S1.** Smoothed current smoking prevalence from fully adjusted model with PC prior with alternative parameters (changing pc.u and pc.alpha from the default value u=1 and α=0.01 to u=0.1,α=0.01). **Figure S2.** Horvitz–Thompson (HT) estimator vs smoothed prevalence in 154 sampled municipalities (model adjusted for age, gender and education). **Figure S3.** Conditional Predictive Ordinate for in-sample municipalities from the fully adjusted model.

**Additional file 2: Supplementary Table S2.** Smoothed prevalence of current (heavy, moderate and light smokers), former smokers and persons who never smoked with 95% credible interval from model adjusted for municipality level age, gender and education. Estimates for 589 municipalities.

**Additional file 3: Supplementary Table S3.** Smoking attributable all-cause mortality per municipality, in absolute number of deaths. Estimates for 589 municipalities.

## Data Availability

All estimates are available as Supporting Information. The microdata that underly the findings of this study cannot be made publicly available due to legal restrictions. These data are however available from Sciensano following the procedures described at https://www.sciensano.be/en/node/55737/health-interview-survey-microdata-request-procedure.
