# Supplementary material for: Variation in smoking attributable all-cause mortality across municipalities in Belgium, 2018: application of a Bayesian approach for small area estimations
Source: BMC Public Health. 2022 Sep 7;22:1699. doi: 10.1186/s12889-022-14067-y (PMC9451124; doi:10.1186/s12889-022-14067-y)
Supplement: Supplementary file 1 — Additional file 1: Technical details ondesign-based estimators.Table S1. Proportion of missing data on smoking by education, age and gender. Figure S1. Smoothed current smoking prevalence from fully adjusted model with PC prior with alternative parameters (changing pc.u and pc.alpha from the default value u=1 and α=0.01 to u=0.1,α=0.01). Figure S2. Horvitz–Thompson (HT) estimator vs smoothed prevalence in 154 sampled municipalities (model adjusted for age, gender and education). Figure S3. Conditional Predictive Ordinate for in-sample municipalities from the fully adjusted model. [file 12889_2022_14067_MOESM1_ESM.docx]

**Online supplementary file 1.**

Technical details on design-based estimators.

Ignoring the sampling weights, the Bayesian smoothing model takes the form:

$$y_{i}| p_{i}=Binomial(m_{i}, p_{i})$$

$$\theta_{i}=\mathrm{Log}\left( \frac{p_{i}}{{1-p}_{i}} \right)= \mathbf{d}_{\mathbf{i}}\boldsymbol{\beta} + \varepsilon_{i} + s_{i}$$

$$\varepsilon_{i} \sim N(0, \sigma_{\varepsilon}^{2})$$

$$s_{i}| s_{j}, j ∊ ne\left( i \right)\sim N(\bar{s}, \frac{\sigma_{s}^{2}}{n_{i}}),$$

where $m_{i}$is the number of observations in municipality $i$, $n_{i}$ is the number of neighbors for area $i$, $\bar{s}$ = $\frac{1}{n_{i}}$ $\sum_{j∊ne(i)} s_{j}$.  $\mathbf{d}_{\mathbf{i}}$= (1, $d_{i1}, d_{i2}\ldots d_{\mathrm{ip}})$ is the vector of the intercept and $p$ covariates corresponding to municipality $i$, and **β** = ($\beta_{0}, \beta_{1}, \beta_{2}\ldots\beta_{p}$) is the coefficient vector.

The unstructured component $\varepsilon_{i}$ is modelled as independent and identically distributed normal variables with zero mean and variance $\sigma_{\varepsilon}^{2}$. Hyperpriors are put on $\boldsymbol{\beta}$, $\sigma_{\varepsilon}^{2}$, and $\sigma_{s}^{2}.$

Table S1. Proportion of missing data on smoking by education, age and gender.

|  | **N complete** | **N missing** | **N(%) missing** |
| --- | --- | --- | --- |
| Low education | 3745 | 1146 | 23% |
| High education | 3932 | 747 | 16% |
| Age <65 | 5895 | 1475 | 20% |
| Age 65+ | 1934 | 449 | 19% |
| Male | 3744 | 930 | 20% |
| Female | 4085 | 994 | 20% |


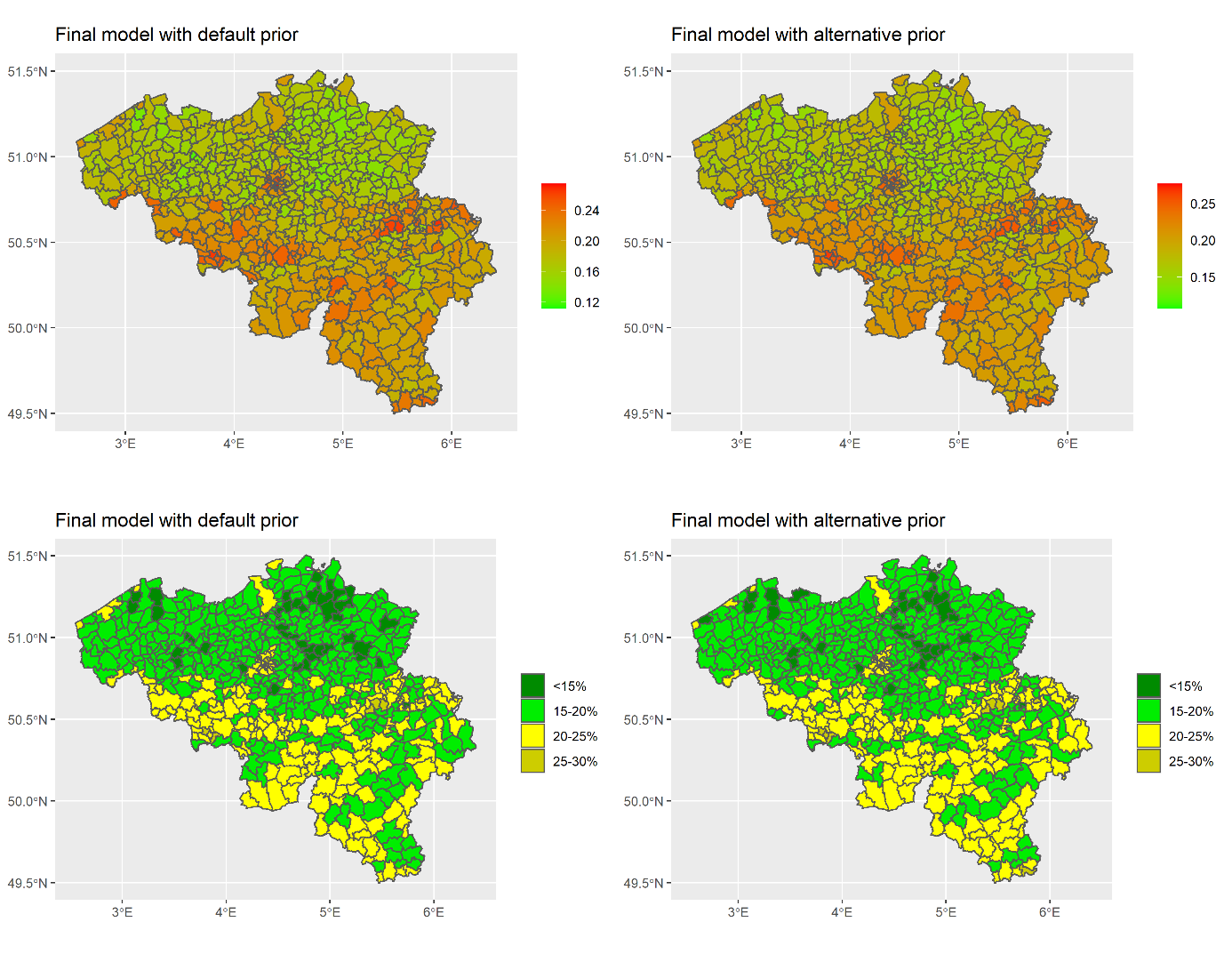


Figure S1 Smoothed current smoking prevalence from fully adjusted model with PC prior with alternative parameters (changing pc.u and pc.alpha from the default value u=1 and α=0.01 to u=0.1,α=0.01)


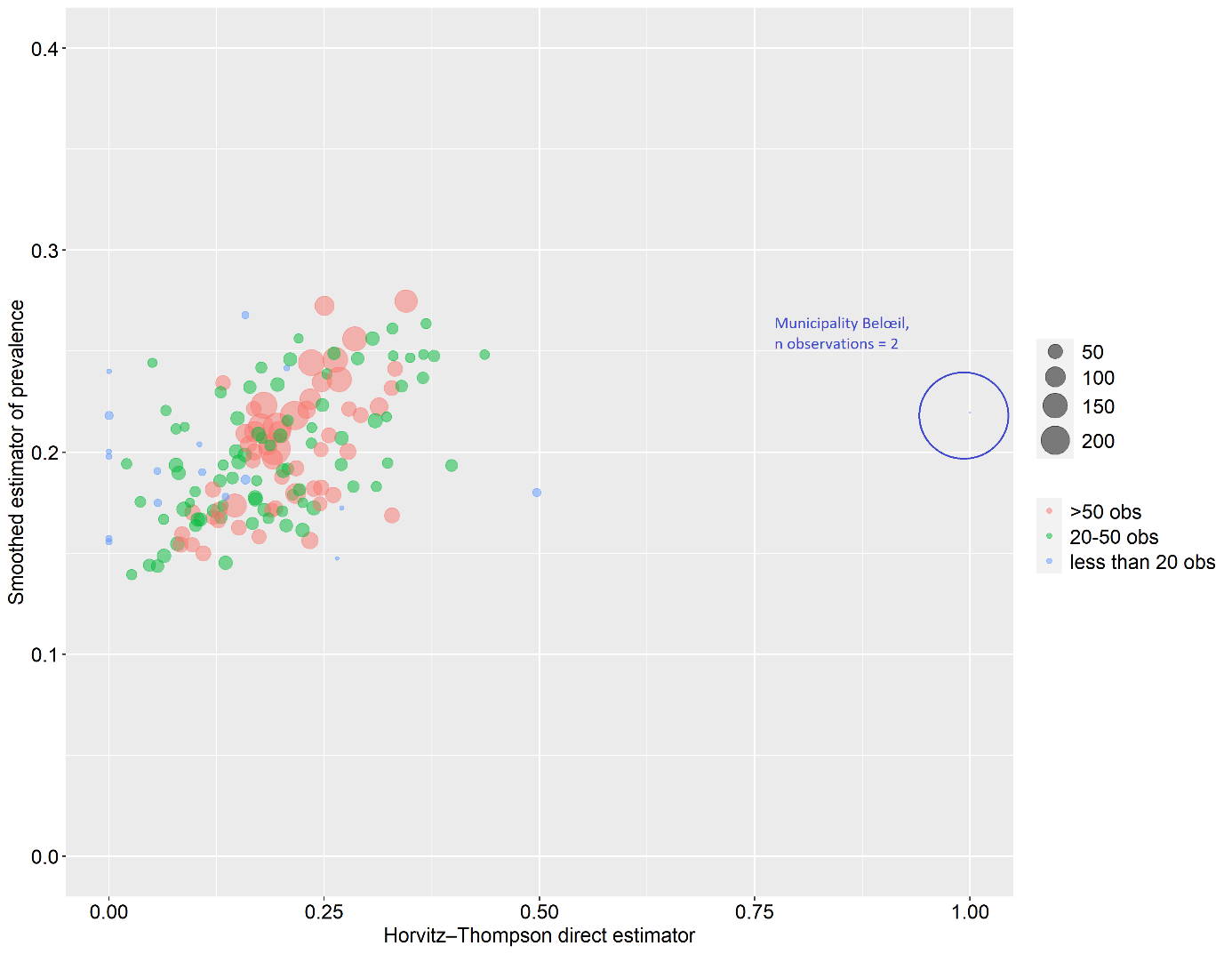


Figure S2 Horvitz–Thompson (HT) estimator vs smoothed prevalence in 154 sampled municipalities (model adjusted for age, gender and education)


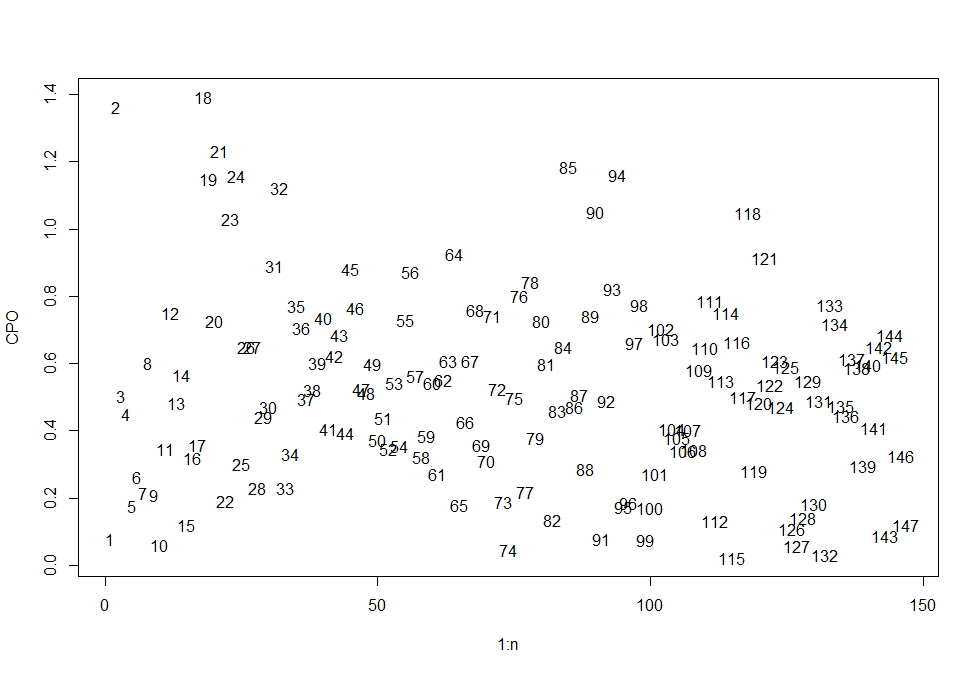


Figure S3 Conditional Predictive Ordinate for in-sample municipalities from the fully adjusted model
